# Gut Hormones in Health and Obesity: The Upcoming Role of Short Chain Fatty Acids

**DOI:** 10.3390/nu13020481

**Published:** 2021-01-31

**Authors:** Habeeb Alhabeeb, Ali AlFaiz, Emad Kutbi, Dayel AlShahrani, Abdullah Alsuhail, Saleh AlRajhi, Nemer Alotaibi, Khalid Alotaibi, Saad AlAmri, Saleh Alghamdi, Naji AlJohani

**Affiliations:** 1 Research Center, King Fahad Medical City—KFMC, Riyadh 11525, Saudi Arabia; aalfaiz@kfmc.med.sa (A.A.); ekutbi@kfmc.med.sa (E.K.); daalshahrani@kfmc.med.sa (D.A.); aalsuhail@kfmc.med.sa (A.A.); salamri@kfmc.med.sa (S.A.); saghamdi@kfmc.med.sa (S.A.); 2 Family Medicine, King Fahad Medical City—KFMC, Riyadh 11525, Saudi Arabia; salrajhi@kfmc.med.sa; 3 College of Medicine, Shaqra University, Shaqra 11961, Saudi Arabia; ne.alotaibi@su.edu.sa (N.A.); kalruwis@su.edu.sa (K.A.); 4 Obesity, Endocrine, and Metabolism Center, King Fahad Medical City—KFMC, Riyadh 11525, Saudi Arabia; njaljohani@kfmc.med.sa

**Keywords:** obesity, gut hormones, short chain fatty acids, diabetes, overweight, food intake, appetite, glucagon-like peptide-1, peptide tyrosine tyrosine, neuropeptide Y

## Abstract

We are currently facing an obesity pandemic, with worldwide obesity rates having tripled since 1975. Obesity is one of the main risk factors for the development of non-communicable diseases, which are now the leading cause of death worldwide. This calls for urgent action towards understanding the underlying mechanisms behind the development of obesity as well as developing more effective treatments and interventions. Appetite is carefully regulated in humans via the interaction between the central nervous system and peripheral hormones. This involves a delicate balance in external stimuli, circulating satiating and appetite stimulating hormones, and correct functioning of neuronal signals. Any changes in this equilibrium can lead to an imbalance in energy intake versus expenditure, which often leads to overeating, and potentially weight gain resulting in overweight or obesity. Several lines of research have shown imbalances in gut hormones are found in those who are overweight or obese, which may be contributing to their condition. Therefore, this review examines the evidence for targeting gut hormones in the treatment of obesity by discussing how their dysregulation influences food intake, the potential possibility of altering the circulating levels of these hormones for treating obesity, as well as the role of short chain fatty acids and protein as novel treatments.

## 1. Introduction

Historically, humanity has dealt with countless famines, where the scarcity of food resulted in starvation and significant loss of life. However, today we are facing a different weight associated epidemic, obesity [1]. Obesity is a complex condition that, in simple terms, is caused by a chronic imbalance between energy intake and energy expenditure, and is defined as a body mass index greater than 30 kg/m^2^. Since 1975, the level of obesity worldwide has almost tripled [2]. According to World Health Organization data, 1.9 billion people worldwide are overweight, with 650 million of those considered obese [2]. Data from the Health Survey for England carried out in 2017 revealed that in the United Kingdom, 28.1% of adults are obese, and predict that this number would rise to 48% of the population by 2030 [3]. Furthermore, in the United States of America, 42.4% of adults were considered obese in 2017–2018 [4].

## 2. Obesity Related Diseases

Non-communicable diseases, which are associated with obesity, are the leading cause of death worldwide [5]. Obesity is linked to an increased risk of developing a variety of these diseases such as type 2 diabetes mellitus (T2DM), coronary heart disease, stroke and certain types of cancer [6,7,8,9]. The predicted increase in prevalence of obesity by 65 million people in the USA and 11 million in the UK is expected to lead to an additional 8·5 million cases of diabetes, 5.7–7.3 million cases of heart disease and stroke and 492,000–669,000 additional cases of cancer in both countries by 2030 [10]. Furthermore, conservative estimates of the burden of disease indicators in children predict that 20,000 obese children in Europe have T2DM, a further 400,000 have impaired glucose tolerance, and over 1 million are likely to show a range of indicators for cardiovascular disease, including hypertension and raised cholesterol levels and have three or more indicators of metabolic syndrome. Finally, over 1.4 million children may have early stages of liver disorder linked with non-alcoholic fatty liver disease [11]. While these conditions have typically been characteristic to Western countries, their prevalence is now rising in developing countries, leading to a double burden of non-communicable diseases and undernutrition. Rapid action is required to stop this global rise in obesity [1].

In addition to significantly impacting patients’ quality of life, the obesity epidemic places a huge burden on health services [10]. A systematic review on the economic burden of obesity worldwide by Withrow and Alter (2011) found that medical expenses for obese individuals can be 6%–45% higher than for their normal weight counterparts. Globally, between 0.7% and 2.8% of a country’s total health expenditure is associated with obesity related costs. Estimates suggest that in the UK, the National Health Service spent £6.1 billion on overweight and obesity related ill-health in 2014–2015, which is expected to rise to £9.7 billion by 2050 [12]. There was also a reported wider societal cost of £27 billion. Furthermore, in 2016 the US spent $480.7 billion on direct obesity-related healthcare costs, and there was an estimated indirect societal cost of $1.24 trillion [13].

The remarkable prevalence of obesity and its consequences on overall health have led to calls to identify the root causes of obesity and potential treatments. At a societal level, obesity can be considered as a product of the modern Western lifestyle, resulting from obesogenic environments with the wide availability of convenient high-reward and calorie-dense foods, excessive portions and lack of daily exercise [14]. Research has revealed that obesity can also have a small genetic component. This can take the form of a monogenic disorder in more extreme cases, such as possessing mutations in genes involved in the leptin/melanocortin axis [15], but is more often seen as a polygenic disorder affecting many different genes. In these cases, most of the genes involved are linked with appetite regulation [16]. This link between obesity and appetite regulation highlights the importance of the gut–brain axis in body weight homeostasis, through the lens of satiation [17]. In principle, food ingestion in humans results in gastric distension and secretion of hormones associated with satiety, leading to a short-term reduction in food intake [18,19,20].

In the first instance, treatment for obesity involves lifestyle interventions to change dietary and physical activity patterns to promote weight loss. However, these interventions are often unsuccessful [21,22,23]. The National Institute of Care and Excellence (NICE) states that if lifestyle changes are found to be ineffective, pharmacological treatments may be considered [24]. Despite the urgency for effective medical treatments for obesity, to date the options are limited. The sole drug treatment available in the UK, Orlistat, does not yield impressive results regarding long-term weight loss [25], and is associated with gastrointestinal side effects [26]. If these methods have failed, health care practitioners may then discuss weight loss surgery with patients. The only treatment that drastically improves patients’ weight management in a sustainable way is the Roux-en-Y gastric bypass (RYGB) surgery. However, this is a costly, invasive procedure with side effects, that may not always be effective for everyone, for example in elderly patients [27,28]. Furthermore, up to 20% of patients who undergo RYGB experience significant weight regain [29]. Intriguingly, weight loss post-surgery also coincides with an altered pattern of secretion of gut hormones that control appetite [30,31,32]. Collectively, both genetic evidence and data from patients who have undergone bariatric surgery highlight the contribution of appetite control to obesity through the role of satiety-inducing gut hormones [15,27,32,33,34].

## 3. Appetite Regulation

Appetite in humans is carefully regulated by a complex system of neural signals, hormones and external stimuli, which manage the intake of food [33]. This regulation system can be broken down into central regulation involving the arcuate nucleus of the hypothalamus, and peripheral regulation via hormones which are released in the gut or elsewhere. This system requires a delicate balance of signals to function optimally, and its dysregulation can lead to imbalances in energy input compared to output, eventually leading to weight gain and obesity [35]. Appetite regulation can have both homeostatic and hedonic elements [35,36]. Homeostatic appetite control involves the regulation of energy intake needed for bodily functioning and to keep body weight static. However, homeostatic regulation of energy intake has evolved over a time when food was often in short supply, thus having very strong orexigenic signalling. Therefore, it may not be optimal for our current obesogenic environment, where there is an abundance of food [37]. Hedonic eating on the other hand, refers to eating for pleasure, which most often involves cravings for foods high in fat and sugar, which are linked to obesity [38]. This type of eating involves dopaminergic pathways in the midbrain linked with addiction behaviours, and has the power to override homeostatic satiety signals, causing a person to eat even when they are full [38].

## 4. Gut Hormones and Appetite Regulation

In humans the gut is the largest endocrine organ and is a highly metabolically active system. It plays an integral part in energy homeostasis, producing around 100 bioactive peptides and expressing more than 30 genes encoding gut hormones [39]. These gut hormones are differentially secreted by the enteroendocrine cells in response to nutrient fluctuation, creating the fasting and fed secretory profile [40]. Gut hormones along with adipose tissue secreted peptides are critical for the homeostatic control of body mass through regulating food intake and energy expenditure [41]. These hormones mediate their effects directly via acting on areas in the brain lacking blood–brain barrier or indirectly via stimulating the vagal afferent neurons [42]. 

The pancreatic polypeptide (PP-fold) family are a group of endocrine peptides, including peptide tyrosine tyrosine(PYY), neuropeptide Y (NPY) and pancreatic polypeptide (PP) [17]. These are 36 amino acid peptides which share a common sequence and structure, the PP-fold. PYY is secreted by the intestinal L-cells in the mucosa of the distal ileum and colon after eating a meal, in proportion to the quantity of food consumed [43,44]. PYY secretion is also differentially triggered by the macronutrient content of a meal, with high protein meals increasing the PYY serum levels more than meals high in carbohydrate and fats [45]. PYY exerts anorexigenic functions through interaction with the selective Y_2_ receptor agonist by interacting with the vagus nerve and inhibiting NPY neurons and promoting proopiomelanocortin (POMC) neurons via the hypothalamus [46,47]. This results in inhibition of gastric motility and emptying, and suppressing pancreatic secretion [48,49]. PP is secreted by a group of cells localized in the periphery of the pancreatic islets of Langerhans in response to food ingestion [50,51,52,53]. PP exerts its anorectic effects via all Y receptors, but has the highest affinity to the Y_4_ receptor [54]. Like the PYY hormone, the level of PP secreted is proportional to the calorie and macronutrient composition of a meal. However, unlike PYY, higher levels of PP are secreted in response to consumption of a high-fat diet [55].

Another category of gut hormones are the proglucagon-derived peptides including glucagon, glucagon like peptide-1 (GLP-1), GLP-2, and Oxyntomodulin (OXM). All these peptides are created from proglucagon which is differentially processed into glucagon in the pancreas, and GLP-1/2 and OXM in the brainstem and the L-cells of the small intestine [56,57,58]. These proglucagon-derived peptides act through the GLP-1 receptor, which is expressed in the brain, gut, and pancreas [59,60,61]. OXM and GLP-1 are co-secreted in response to food consumption and act as a satiety signal, limiting food intake [60,62], whereas GLP-2 is important for the proliferation of crypt cells in the gut [63,64,65]. Glucagon, a 29 amino acid peptide, is secreted from α-cells in the pancreas when circulating glucose levels are low. Despite glucagon having an opposing effect on glucose homeostasis compared to GLP-1, with glucagon increasing circulating glucose and GLP-1 stimulating insulin release in response to food intake, they both have an anorectic effect, reducing food intake [66].

GLP-1 is a 30 amino acid peptide which acts as an incretin, meaning it reduces circulating levels of glucose via stimulating insulin secretion [67]. It also acts by inhibiting glucagon secretion, thus reducing endogenous glucose production, reducing food intake and slowing gastric emptying [17,49,68]. It is speculated that the satiating effects of GLP-1 are brought about by interaction with NPY/Agouti-related peptide (AgRP) and POMC neurons, however some research suggests that these effects are caused by action on other anorexigenic hormones [7]. The ability of GLP-1 to control insulin responses can be important for improving the glycaemic control in patients with type 2 diabetes [69].

Oxyntomodulin (OXM) is a 37-amino acid peptide, which is secreted in response to food intake, in proportion to the calorie content of the meal [62]. It is composed of the 29 amino acids in glucagon, along with an eight-amino acid C-terminal extension. This hormone is a dual agonist with its anorectic influence thought to be mainly mediated by GLP-1 receptor, while the weight-lowering effect of OXM is mediated by the glucagon receptor [70]. OXM is a potent anorectic hormone; despite having a much lower affinity for the GLP-1 receptor than GLP-1, it still has the same effect as GLP-1 on satiety [60]. Unlike GLP-1, its effect on energy intake does not involve glucose homeostasis [71]. External supplementation with OXM or GLP-1 results in increased energy expenditure and decreased food intake [57,71,72,73]. GLP-2 is also secreted by the L-cells in response to food consumption, however, it does not affect the food intake [74,75]. 

Cholecystokinin (CCK) is released from intestinal L-cells in response to ingestion of fat and protein through mechanisms involving the G-protein-coupled receptors GPR40 and CaSR [76,77]. While its main role is in the digestion of these nutrients by stimulating the production of bile and pancreatic and gastric secretions, it also delays gastric emptying and suppresses food intake. It is thought that CCK acts directly on vagal afferent neurons which terminate in the nucleus of the solitary tract (NTS) [78]. These neurons are first stimulated in the intestine in a paracrine manner by locally acting CCK, and as circulating levels increase, there is hormonal stimulation of vagal afferent neurons lining the stomach [78]. CCK activates the cholecystokinin receptor type A, which is thought to play a role in mediating the secretion of GLP-1 and PYY, and the inhibition of ghrelin after food intake [79,80].

**Figure 1:**
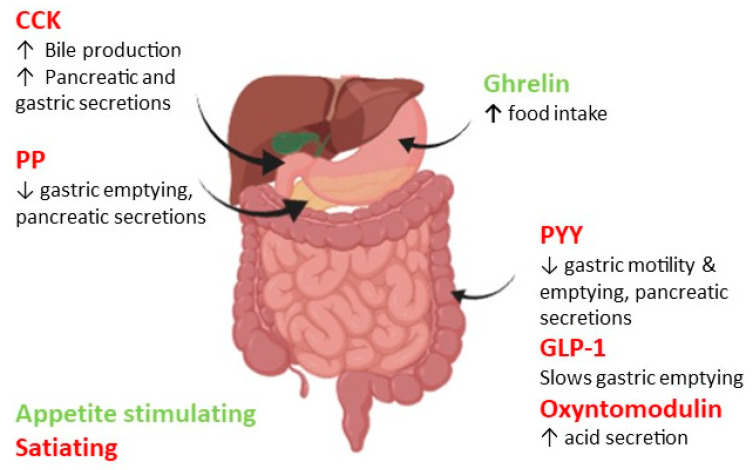
Summary of gut hormones involved in appetite regulation. Ghrelin, the orexigenic hormone in green, is released from the stomach and stimulates food intake. The anorexigenic hormones shown in red (Cholecystokinin (CCK), Pancreatic Polypeptide (PP), Peptide YY (PYY) and Glucagon-Like Peptide 1 (GLP-1)) are released from various organs in the gastrointestinal system and work together to decrease food intake by slowing gastric motility, emptying and reducing acid and pancreatic secretions.

Ghrelin, a 28 amino acid peptide, also known as the hunger hormone, is the only gut hormone that increases appetite, thus showing an orexigenic effect. The hormone is secreted by the P/D1 enteroendocrine cells in the gastric fundus during the fasting state and decreases after food intake [81], leading to an increase in energy intake and appetite [82,83]. Postprandial levels of ghrelin decrease proportionally to the caloric load and macronutrient composition of the meal, with fat being least effective at lowering ghrelin levels while protein is the most effective [82,84]. The acylation of ghrelin by ghrelin O-acyltransferase (GOAT) is necessary for the hormone’s effects on food intake [82]. Ghrelin acts by stimulating NPY/AgRP expressing neurons and inhibiting POMC expressing neurons [82]. This hormone also promotes weight gain and adiposity by acting on neurons in the paraventricular nucleus of hypothalamus [85]. Low dose pre-prandial infusions of ghrelin have been shown to increase ad libitum food intake obese participants, while high doses increased food intake in both lean and obese groups compared to when participants received saline infusion [83]. However, it is important to note that this double blinded randomised controlled trial (RCT) only included 24 participants, although infusions were administered in a random order throughout the trial. A schematic diagram summarizing the main roles of anorexigenic and orexigenic peptides on appetite regulation is shown in Figure 1.

## 5. Signalling Pathways of Appetite Regulation

At the central nervous system level, appetite is controlled by the balance between anorexigenic (appetite suppressing) and orexigenic (appetite stimulating) signalling pathways. The peripheral cues that instruct the neuronal responses are provided through humoral and neuronal connections, such as the bloodstream and the vagus nerve [36,42]. As well as the homeostatic regulation of appetite in the central nervous system, there is also an influence on control of hedonic food intake in some parts of the brain, such as the ventral tegmental area (VTA) and the nucleus acumbens (NA), which are part of the mesolimbic reward system, a dopaminergic pathway in the brain [85].

The centre for appetite and energy homeostasis is the hypothalamus, specifically, the arcuate nucleus (ARC). Anatomically, the ARC is found close to a highly permeable part of the blood brain barrier, allowing it to be directly exposed to the bloodstream and hence act as the primary sensor for peripheral metabolic signals [35]. In the ARC, appetite is regulated through the activity of two neuronal subtypes. The first subtype respond to orexigenic neuropeptides, such as NPY and AgRP, whereas the second type respond to anorexigenic neuropeptides including POMC, which is co-expressed with cocaine- and amphetamine-regulated transcript (CART) [35,36]. These neurons are either inhibited or stimulated via signals from these peripheral hormones and food intake [35,36]. AgRP works by antagonistic binding to melanocortin 3 and 4 receptors (MC3/4R), and thus increases food intake [86], while NPY stimulates food intake via the NPY Y1 and Y5 receptors. Furthermore, NPY/AgRP also increase appetite by inhibiting POMC neurons [85]. Upon ingestion of food POMC/CART neurons are stimulated and POMC is cleaved into α-melanocyte-stimulation hormone, which activates MC3/4R, and in turn leads to decreased food intake [85]. Intriguingly, dysregulation of post-transcriptional processing of POMC as well as mutations in the melanocortin receptors results in severe early-onset obesity, hyperphagia, and reduced energy expenditure [87,88].

Another key area for appetite regulation is the brainstem, and specifically the NTS which is innervated by the vagus nerve, creating a connection between the gastrointestinal tract and the brain [89]. Neurons in the NTS modulate feeding behaviours by exciting areas of the brain such as the lateral parabrachial nucleus (LPN) [90]. These neurons in the NTS are activated by duodenal rather than gastric distention, as well as CCK which is released into the duodenum in response to fat and protein intake [89]. NTS neurons are also exposed to the humoral peripheral signals and secrete GLP-1, NPY, and POMC as a response [91]. Activation of either the NTS or ARC results in altered secretion of neuropeptides, or activation of second order neurons.

Hormones interact differentially with neurons at different centres to induce their effects. At a molecular level PYY acts through engaging the Y2 receptors on the NPY ARC neurons reducing their activity and thus pushing the balance towards anorexia [54]. Intriguingly, a genetic single nucleotide polymorphism that affects the binding of PYY to its receptor has been associated with increased food intake and body mass [92]. PYY and GLP-1 can also induce anorexia via interacting with the vagus nerve and the promoting the activity of POMC neurons that secrete anorectic peptides [93]. PP acts directly on the CNS through Y4 receptors in the brainstem and hypothalamus to reduce appetite [94]. In rodent models, peripheral infusions of PYY and have been shown to decrease acute energy intake by up to 45% in non-obese NIH/Swiss mice 60 min post-infusion, while reducing body weight dose-dependently in obese rodents over a four-week period of daily infusions, including ob/ob mice, diet-induced obese mice and nondiabetic Fatty Zucker rats [95].

**Figure 2:**
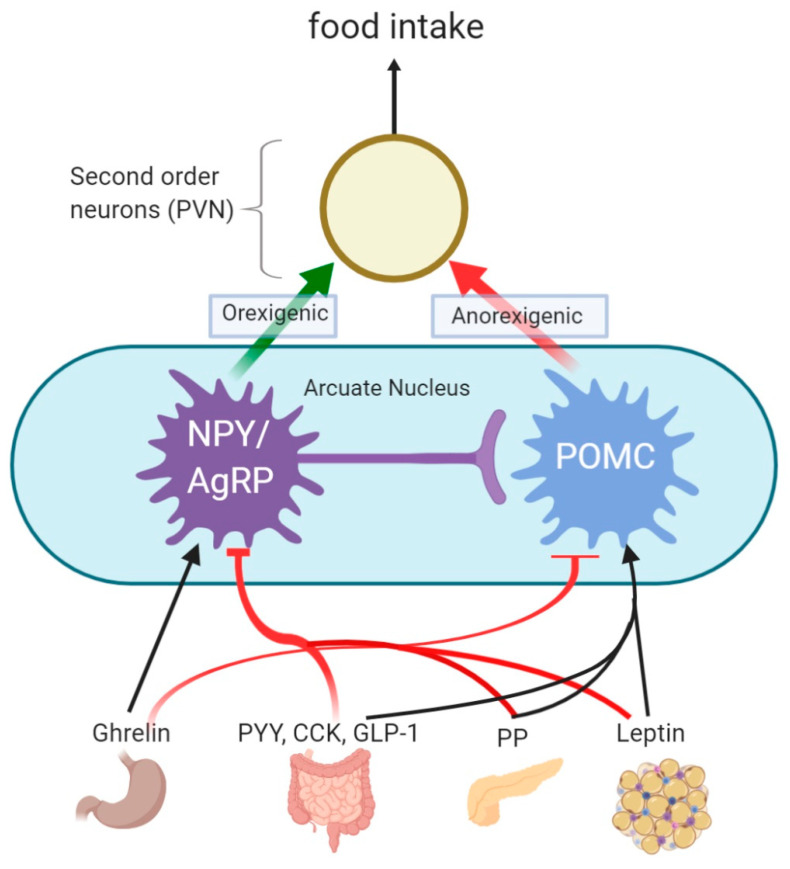
Signalling pathways involved in appetite regulation. This schematic diagram shows the effect of hormones on neuronal appetite signalling in the brain. Ghrelin is released from the stomach, stimulates (black arrow) the orexigenic Neuropeptide Y (NPY)/Agouti-related Peptide (AgRP) and inhibits (red lines) anorexigenic proopiomelanocortin (POMC) neurons in the arcuate nucleus. While Leptin from adipose tissue, pancreatic polypeptide (PP) from the pancreas and peptide YY (PYY), Cholecystokinin (CKK) and glucagon-like peptide 1 (GLP-1) from the intestines stimulate POMC while inhibiting NPY/AgRP. This then feeds into the second order neurons in the Paraventricular nucleus of hypothalamus (PVN) to influence food intake.

Leptin, although not a gut hormone, also plays an important role in signalling in appetite regulation. It is a long-term satiety hormone which is secreted by white adipocytes in proportion to the size of the cells [35]. A decrease in circulating leptin leads to an increase in appetite, and therefore a loss of adipose tissue leads to an increased appetite and acts as the body’s attempt to regain the lost weight. High levels of circulating leptin inhibits AgRP/NPY and stimulates POMC/CART leading to stimulation of anorexigenic neurons [96]. A schematic diagram showing central regulation of anorexigenic and orexigenic signalling is shown in Figure 2.

## 6. The Gastrointestinal Tract and Appetite Regulation

Motilin, a hormone released from gastrointestinal endocrine cells in the fasting state, is a key hunger signal involved in regulating the migrating motor complex (MMC). In the fasting state, the gastrointestinal tract experiences the MMC cycle, altering between periods of quiescence and periods of contractions, which move from the stomach to the distal ileum [97]. In total, this cycle lasts an average of 90–120 min, comprised of four phases. Phase I is the quiescent stage, with slow waves, but no contractions, phase II involves random contractions over 10–15 min, and phase III is characterized by a sudden onset of regular contractions that can start in the stomach or the small intestines for 5–10 min [97]. The feeling of hunger is induced by phase III of the MMC cycle and circulating levels of motilin have been found to correlate with hunger scores during this phase. Phase IV involves the rapid decrease in contractions, after which the cycle restarts at phase I if food has not been ingested. It is thought that increased circulating levels of PP postprandially leads to decreased circulating levels of motilin, and thus can alter the MMC cycle [97,98]. Additionally the distension of the stomach after food intake interrupts MMC activity in the stomach and duodenum [98].

A trial of 16 morbidly obese (BMI > 40 kg/m^2^) participants found that in a fasted state, obese patients had significantly higher motilin levels compared with healthy controls during the MMC cycle, but tended to lack the motilin peak prior to phase III. This peak in motilin levels in necessary to trigger hunger signals, which may explain why these obese patients also had lower hunger scores during phase III [99]. In this trial, infusions of erythromycin, a motilin receptor agonist, were administered to patients over a 20 min period, and began 20 min after phase III MMC at two timepoints, six months prior to undergoing RYGB and one year after the surgery. Hunger levels were scored every 5 min during this infusion and phase III contractions were measured. Antroduodenal contractions were measured pre-RYGB, and after surgery contractions were measured in the constructed Roux limb. Administration of erythromycin restored both gastric phase III contractions and hunger scores in obese patients, indicating that the motilin receptor in obese patients responded to the exogenous administration of erythromycin but fails to respond to endogenous motilin release. The authors suggested two possible explanations for this attenuated response towards motilin. Firstly, this may be due to a lack of motilin fluctuation or secondly, desensitisation of the motilin receptor towards motilin. After undergoing RYGB, motilin levels reduced to a similar concentration as the healthy volunteers, but erythromycin no longer had the ability to induce phase III contractions [99]. Future studies will further reveal the role of gut motility and MMC on changes in gut hormones and appetite regulation.

## 7. Relationship between Gut Hormones and Obesity

As discussed previously, gut hormones are critical in the regulation of appetite and energy intake in humans, and obesity is the consequence of prolonged excess energy intake and appetite dysregulation. As expected, gut hormones can be differentially regulated in obese individuals [17,93], although, it is not always possible to know whether this is a cause or consequence of obesity [66]. However, there are some circumstances, for instance in genetic forms of obesity such as leptin deficiency or Prader–Willi syndrome, which are characterized by increases in orexigenic hormones and decreases in anorexigenic hormones, which partly mediate the characteristic overeating of these conditions [34,100,101].

In polygenic obesity, where multiple genes are implicated, single nucleotide polymorphisms (SNPs) in the fat mass and obesity-associated (FTO) gene have been identified as having the biggest impact of a single polymorphism in increasing the risk of obesity [102]. This is due to people with this risk variant of the gene having higher circulating levels of ghrelin, causing them to feel hungrier [103]. A recent UK cohort study found that 16% of the adult population are homozygous for this risk allele and have a 1.67-fold increased risk of being obese than those who do not possess the risk allele [101].

There is mixed evidence surrounding the link between obesity and levels of PYY and GLP-1, with some studies showing that those who are obese have lower circulating levels of these hormones than those who are a healthy weight, while others have not found this [49]. Lower levels of PYY in have been observed in both fasting and fed states in obese children and adults, suggesting a complex deviation in the homeostatic regulation of the hormone. It has been suggested that this is due to abnormalities in the synthesis, release or clearance of PYY [104]. Moreover, studies showed that after RYGB circulating levels of both PYY and GLP-1 are significantly increased, strongly correlating the post-surgery weight loss with the hormone levels [37].

Other gut hormones altered in obese individuals include PP and ghrelin. Fasting levels of appetite hormones in 38 obese children and 35 lean children were assessed and PP concentrations were found to be lower in obese children compared to their lean counterparts (29 fmol/mL (19–58 fmol/mL) vs 73 fmol/mL (51–137 fmol/mL), respectively). At the one-year follow-up, PP levels had significantly increased in children who had lost weight [105]. Intriguingly, patients with Prader–Willi syndrome experience reduced levels of secreted PP and one in three display high levels of fasting ghrelin [106,107]. This may play a role in the hyperphagia and obesity, a distinctive feature of Prader-Willi syndrome, suggesting a strong correlation between the hormone and appetite regulation [107].

In a randomized cross-over trial looking at ghrelin response to food intake with 15 obese and 12 lean young Chinese adults; obese subjects demonstrated impaired ghrelin suppression after a high fat meal, leading to reduced satiety [70]. This is in line with previous findings that obese people have lower pre-prandial ghrelin and also have a reduced suppression of ghrelin postprandially [84]. Circulating ghrelin levels can also be increased by physiological stress and is thought to be involved in stress-induced overeating which contributes to overweight and obesity [108]. Furthermore, as well as this dysregulation of and desensitization to gut hormones in obesity, weight loss can lead to alterations in the level of secretion of some hormones, such as GLP-1 and CCK, to reduce satiety signalling and promote increased food intake, which may partly explain why weight regain is so common [109].

## 8. Gut Hormones in the Treatment of Obesity

Due to the lack of success of lifestyle interventions in reducing obesity, and the increasing prevalence of obesity and related economic burden, researchers are beginning to seek alternative potential treatments for obesity. Until recently, the only licensed drug for the treatment of obesity was Orlistat, which inhibits pancreatic lipase thus preventing fat absorption [93]. However, this treatment has modest results, with an average of only 2.93% sustained weight loss according to a meta-analysis in 2007, and there is a need to develop more effective pharmacotherapies for obesity [25]. Additionally, gastrointestinal side effects are common in patients taking Orlistat [110]. One potential alternative approach is to take advantage of the role gut hormones play in regulating appetite. Currently, RYGB has the most sustainable impact on weight reduction and maintenance, and this is thought to be in part due to a coordinated response from PYY, GLP-1 OXM, and ghrelin, which leads to increased satiety signalling after surgery [37].

Administration of exogenous PYY has been suggested as a potential obesity treatment. This approach is promising as obese individuals retain their sensitivity to the anorexigenic effects of PYY intravenous injection in both rats and humans [46,104]. In a placebo-controlled crossover study of 24 subjects, intravenous infusions of PYY in obese and non-obese individuals reduced the food intake, with no reports of side effects, by 30% and 32%, respectively; suggesting that the hormone acts in the same way irrespective of body mass [104]. However, it is important to note that most of this reduction was seen in the first 12 h after infusion. This effect may have been partly mediated by the reduction in plasma ghrelin after PYY infusions. Further to this, infusions of PP, in amounts sufficient to achieve plasma concentrations similar to normal postprandial levels, leads to delayed gastric emptying and reduced acute food intake, weight gain and glycaemic indices in rodent models [94,95].

Despite the potential of PYY infusions for the reduction of food intake, intravenous infusions may not be the most practical method of delivery as it would need to be administered regularly. As an alternative, in a double blinded RCT with 133 obese patients a nasal spray was used to deliver either a placebo, or PYY in doses of 200 μg or 600 μg for 12 weeks. Researchers also tested the tolerability of PYY at higher doses, and found that the incidence of nausea increased at doses of 800 μg, and at doses of 1000 μg, dizziness and palpitations were experienced by some participants. For this reason, 600 μg was considered the maximum tolerable dose. While they found an average reduction in weight of 3.7 kg and 1.4 kg for the 200 μg and 600 μg, respectively, this reduction in weight was not significantly different from the placebo group [111]. However, this proof-of-concept study demonstrates the feasibility of using nasal spray as a route of administration.

GLP-1 receptor agonists have already been used to develop treatments for T2DM, but its role in satiety signalling suggests it may also be an effective treatment in obesity without diabetes [93]. Intravenous administration of GLP-1 in humans can reduce food intake in a dose dependent manner. A meta-analysis examining the effect of GLP-1 infusions on food intake showed a reduction in ad libitum energy intake by 13.2% and 9.3% in lean and obese individuals, respectively. However, side effects such as nausea and vomiting have been reported [73]. Liraglutide, a GLP-1 receptor agonist was originally developed for type-2 diabetes, however, due to its influence on weight loss, it has recently been approved as a therapy for obesity as a daily subcutaneous injection at doses of 3.0 mg. A recent meta-analysis of five RCTs found that daily administration of liraglutide combined with dietary changes and physical activity led to 4–6 kg weight loss, with a greater proportion of patients achieving at least 5–10% weight loss compared with the placebo group [112]. However, nausea was common, particularly in the first four weeks of treatment, and when compared with other medications for obesity, liraglutide has the highest discontinuation rates due to adverse effects [112]. Another GLP-1 receptor agonist, semaglutide, which is used to treat type 2-diabetes may also be a useful treatment for obesity irrespective of diabetes status. Unlike liraglutide, semaglutide is only administered on a weekly basis rather than daily, making it less burdensome for the patient. A randomised controlled phase 2 trial of this drug found that doses of 0.2–0.4 mg semaglutide resulted in more weight loss over the course of a year, than daily injections of 3.0 mg liraglutide (11–14% versus 7.8% respectively). In contrast to other obesity medications, weight loss associated with semaglutide continued throughout the year, whereas with many other medications weight loss was concentrated in the early period [113]. Taken together, this evidence indicates semaglutide could be quite an effective obesity treatment in the future. Various phase 3 trials of semaglutide are now underway [114,115,116].

Another hormone being examined as a treatment for obesity is OXM. Self-administered OXM can reduce food intake as well as increase activity-related energy expenditure in overweight and obese adults [25,73]. In a double-blinded RCT including 26 overweight and obese participants, subcutaneous injections of OXM or saline were self-administered three times daily over a four-week period and a weight loss of 2.4 ± 0.4% was observed, compared to 0.5 ± 0.6% in the placebo group [117]. Transient mild nausea was reported for 3% of the OXM injections, compared to 0.2% of saline injections, however this was not found to be statistically significant. While these data support the potential of OXM as an anti-obesity treatment, further research is being carried out to look at the long-term effectiveness and safety of OXM treatment [93].

As increases in circulating ghrelin levels leads to increased hunger, rather than treatment with the hormone itself, research is focusing on ghrelin receptor antagonists which would block ghrelin receptors to reduce hunger signalling. However, the results from research on animal models has produced mixed results thus far [118,119,120]. Another possible therapy being examined is oral CCK receptor agonists [121], CCK receptor agonists are used as administration of CCK itself has been found to cause nausea when administered over long periods [93]. While short term trials of orally administered CKK receptor agonists have shown a dose-dependent reduction in the energy intake of obese individuals over a 24-h period, a 24-week long double-blinded RCT of 700 overweight and obese participants did not find a significant difference in weight loss in those who were administered CCK receptor agonist compared to placebo. However, CCK exerts its effects on appetite via leptin, therefore a combination therapy may be more useful [121,122].

Gut hormones can also act synergistically or even additively to reduce energy intake when administered in combination [48,123]. In a study of 12 healthy male subjects, pre-prandial co-administration of oral PYY and GLP-1 resulted in a decrease in ad libitum energy intake of 21.5% compared to a 12% decrease when either hormone was administered on its own [124]. Co-infusion may allow for the administration of lower doses of hormones, reducing the side-effects associated with high-doses, and potentially mimic the coordinated response linked with RYGB, mentioned above [37]. One combination which seeks to mimic the RYGB-associated alteration in gut hormones is GLP-1, OXM and PYY (known as GOP). A mechanistic study examined the effectiveness of this combination in obese patients with prediabetes or diabetes. Participants were randomized to GOP (*n* = 15) or saline (*n* = 11) infusions for four weeks [125]. These participants were also compared to patients who had undergone RYGB (*n* = 21) and those who followed a very low-calorie diet (VLCD, *n* = 22) as unblinded comparators. The authors found that GOP led to more weight loss (−4.4 kg) versus the saline control (−2.5 kg) after four weeks. While weight loss in the GOP group was less than the RYGB (−10.3 kg) and VLCD (−8.3 kg), the effect on glucose tolerance was similar to that of RYGB. No significant side effects were reported [126]. Another hormone combination in development is a GLP-1-glucagon receptor dual agonist (MEDI0382). In a phase 2a randomized controlled trial of 51 patients with obesity and type 2 diabetes [126], participants received daily injections of either MEDI0382, or a placebo for 41 days. At the end of the treatment period those who were given MEDI0382 had significantly better glucose tolerance and more weight loss than the placebo group (3.84 kg versus 1.70 kg, respectively) [126].

## 9. Short Chain Fatty Acids and Appetite Regulation

Intake of fermentable dietary fibre has been negatively correlated with weight gain and obesity [127,128,129], and shown to induce satiety and reduce body weight in humans [130,131,132]. Short-chain fatty acids (SCFAs) are the main products of saccharolytic fermentation of non-digestible carbohydrates (NDC) by bacteria in the colon [127,133]. The primary SCFAs are acetate, propionate and butyrate and their production depends on the availability of dietary fibre and the diversity of the gut microbiota [134,135]. In addition to recovering energy from undigested fibre, SCFAs have multiple roles in human health [136,137,138,139]. SCFAs’ fermentation and absorption in the colon exert their anorectic effects through inducing the release of satiety-inducing gut hormones, which send anorectic signals to the appetite centres in the brain [140]. SCFAs are the ligands for opioid G protein-coupled receptors, such as the free fatty acids receptors 2 and 3 (FFAR2/3) [141]. The former have a higher affinity for acetate and propionate than butyrate and are found in adipose tissue, pancreas and intestine [142,143]. In addition to GTPase signalling cascades SCFAs can also directly influence gene expression through inhibiting histone deacetylases, thus positively regulating the chromatic architecture [144].

Acetate is likely important for regulating appetite. However, its role is not yet fully understood. On one hand, acetate has been shown to induce the expression of anorectic hormones, such as GLP-1 and PYY, in the hypothalamus [145], thus limiting food consumption. In mouse models, acetate produced in the colon stimulates anorectic signalling in the ARC via the glutamate–glutamine transcellular cycle [145]. However, conflicting results from Perry et al., (2016) indicate that acetate increases the secretion of insulin and ghrelin, leading to obesity [146]. More research needs to be carried out on the potential pathways through which acetate exerts its effects to discern whether it is an orexigenic or anorexigenic factor in appetite regulation.

Propionate is another SCFA which has been shown to reduce food intake via satiety-inducing gut hormones [147]. The anorexigenic effect of propionate is mediated through FFAR2/3 in the L-cells in the intestine, that promotes the secretion of GLP-1 and PYY peptides [147]. Moreover, propionate has been shown to limit the accumulation of lipids in the liver through suppressing gene expression profiles associated with fatty acid synthesis [148,149]. Intriguingly, RYGB, which is principally associated with weight loss and reduction in adipose tissue, has been suggested to increase the levels of propionate in the large intestine [127]. The direct delivery of propionate in the proximal colon results in increased levels of PYY and GLP-1 in response to food intake and reduces both food consumption and weight gain in overweight individuals [147].

Butyrate acts through interaction with the receptor GPR109A, which is associated with the colonic inflammatory response [150], and is also important for maintaining the gut barrier integrity [151]. Additionally, butyrate has been shown to influence weight control through increasing the energy expenditure via direct interaction with the skeletal muscle [152], and triggering lipolysis in the adipose tissue [153].

**Figure 3:**
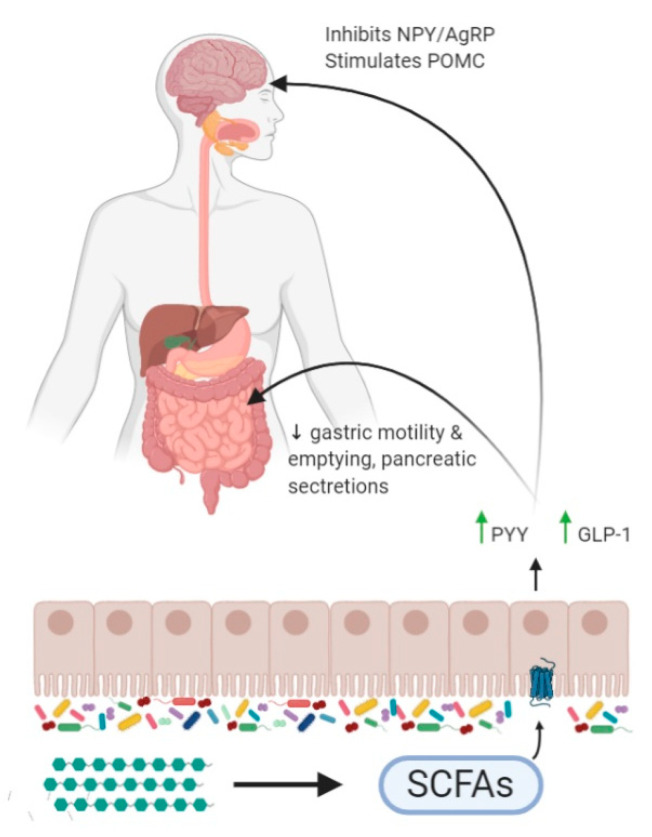
Short chain fatty acids and appetite regulation: Schematic diagram shows that the gut microbiota help to breakdown dietary fibre to produce short chain fatty acids. This in turn stimulates increases in the release of the gut hormones peptide YY (PYY) and glucagon-like peptide-1 (GLP-1) from the L-cells of the colon. The increased levels of GLP-1 and PYY can decrease gastric motility and emptying, as well as reduce pancreatic secretions. The release of gut hormones also inhibits NPY and AgRP neurons, whilst stimulating POMC neurons of the CNS and reduces appetite.

Collectively, SCFAs are the products of microbial fermentation in the colon and have an active role in controlling appetite via increasing the secretion of anorectic gut hormones such as PYY and GLP-1 [154]. As a consequence, increasing levels of SCFAs represents a promising target that could reduce the levels of adiposity and weight in people with obesity. A schematic diagram showing the major pathways that SCFAs can affect appetite regulation is shown in Figure 3.

While this evidence for use of SCFAs in weight management is promising, research in this area is still in the early stages and is comprised mainly of animal studies. It would be important to examine the impacts of administering SCFAs in humans, particularly through methods such as oral or colonic infusions of specific SCFAs, or via dietary fibre. One limitation of an approach involving dietary fibre, is that the breakdown of this fibre and the SCFAs that are produced are influenced by the composition of the gut microbiota, which can vary from person to person. Another important consideration for is that while SCFAs seem to have a role in stimulating appetite regulating hormones, they are also a source of energy. Therefore, when administering SCFAs as an obesity treatment, care must be taken not to increase energy intake to an extent that may negate their effect on satiation [154]. Overall, more research, particularly in humans and on the whole body system, is essential to fully understand the effectiveness and side effects of SCFAs as a treatment for obesity [155].

## 10. The Role of Other Dietary Factors in the Treatment of Obesity

Protein is another dietary component that has been suggested to have a potential therapeutic role in appetite regulation in obesity. A meta-analysis of 49 articles found that acute ingestion of a high protein meal increased satiety shortly after eating [156]. Kohanmoo and colleagues (2020) found protein consumption was linked to a significant decrease in ghrelin (−20; 95% CI: −29, −12 pg/mL; *n* = 25), and a significant increase in CCK (30;95% CI: 17, 43 pg/mL; *n* = 15) and GLP-1 (21; 95%CI: 13, 29 ng/mL; *n* = 25). While levels of ghrelin did decrease after less than 35 g protein, protein intake higher than 35 g altered ghrelin, CCK and GLP-1 more significantly. However, long-term interventions (3 day to 9 months), did not show a significant association between protein and satiety, or gut hormone levels.

There has been much debate on which protein type has the strongest influence on appetite. A systematic review found that whey was the most common protein type used in studies, however, due to small numbers of studies examining other protein types, it was not possible to determine whether there is actually a significant difference in satiating effects. One randomised cross-over study looking at different protein types in 24 overweight and moderately obese young adults consuming three isoenergetic dietary treatments. These treatments varied in protein source between hydrolysed casein, intact casein, and intact whey. After 24 h, there were no differences in appetite regulation or energy expenditure between these groups, apart from lipid oxidation, which was higher in those who consumed intact whey protein source, compared to hydrolysed casein [157].

One side effect of relying on protein consumption to increase satiety is that very high intakes of protein can cause kidney dysfunction by creating a high acid load [158]. The source of protein in the diet is also important, as many protein-rich foods, such as red meat, contain high levels of saturated fat and should be eaten in moderation [158]. 

## 11. Future Directions

While the role of gut hormones in appetite regulation is relatively well understood, the potential of their use as treatments for obesity remains to be elucidated. First, more long-term research needs to be carried out to assess whether the regular administration of gut hormone therapies, such as those mentioned above, have a meaningful impact on obesity, and the maintenance of weight loss. Secondly, different administration routes and doses should also be assessed to minimize side effects as well as the burden on patients. Third, studies should also investigate combining hormone therapies, particularly those that may mimic the hormone alterations seen after RYGB.

There is also the emerging role of SCFAs in obesity therapy to consider. More research is needed in humans on the pathways involved in SCFAs’ role in appetite regulation, the safety of their use, as well as the impact of administering these SCFAs on weight loss in obese individuals. It would also be worthwhile looking at the impact of combinations of anorexigenic SCFAs on weight loss. This research would need to be carried out before they could be included in obesity treatments.

## 12. Conclusions

Due to the rising prevalence of obesity worldwide, there is an unmet need for innovative, minimally invasive and well-tolerated treatments. As discussed, gut hormones play an integral role in the regulation of food intake, and thus, a variety of evidence suggests that the deregulation of and desensitisation to gut hormones may be one of the causative agents of obesity. Supplementation of gut hormones, such as PYY, GLP-1, and OXM, as well as antagonists of ghrelin, have been shown to significantly improve the weight loss outcomes of obese patients, while maximum benefits may be reaped by Administering combinations of gut hormones, particularly those that mimic the gut hormone profile seen after RYGB. Previous research mainly focused on intravenous administration of these treatments, but oral and nasal therapies are also being developed which would provide more convenient treatments. As an alternative approach, SCFAs have the potential to be highly beneficial in the weight management in obese individuals, due to their ability to improve the expression levels of PYY and GLP-1, thus increasing satiety and reducing energy intake. Contrary to RYGB, hormone or SCFA treatment is far less invasive and carries minimal risks to the patient’s life. Additionally, patients are more likely to address obesity if the treatment is administered orally or intravenously, rather than proceed with a life altering surgical operation such as RYGB. Overall, the use of hormone therapy or SCFAs present highly desirable approaches for obesity treatment, but further trials in humans with longer follow-ups are required to assess the long-term effectiveness of these approaches.

## Data Availability

Not applicable.

## Abbreviations

T2DM	Type 2 diabetes mellitus
NICE	The National Institute of Care and Excellence
RYGB	Roux-en-Y gastric bypass
VTA	ventral tegmental area
NA	nucleus acumbens
ARC	arcuate nucleus
AgRP	Agouti-related peptide
POMC	proopiomelanocortin
CART	Cocaine and amphetamine-regulated transcript
MC3/4R	melanocortin 3 and 4 receptors
NTS	nucleus of the solitary tract
LPN	lateral parabrachial nucleus
CCK	cholecystokinin
GLP-1	Glucagon-like peptide-1
PYY	peptide tyrosine tyrosine
PP	Pancreatic polypeptide
PVN	Paraventricular nucleus of hypothalamus
PP	pancreatic polypeptide
NPY	Neuropeptide Y
OXM	Oxyntomodulin
GOAT	Ghrelin O-acyltransferase
RCT	Randomised Controlled Trial
SNPs	single nucleotide polymorphisms
FTO	fat mass and obesity-associated
SCFAs	Short-chain fatty acids
NDC	non-digestible carbohydrates
FFAR2/3	free fatty acids receptors 2 and 3
